# Amalgam as a Filling-Material

**Published:** 1892-07

**Authors:** William P. Cooke


### AMALGAM AS A FILLING-MATERIAL.[1]

BY WILLIAM P. COOKE, D.M.D.

It is now nearly sixty years since this material was introduced to the dental profession. The history of its use is full of interest. From its first introduction into this country, in New York City in 1833, by the Croucours, who were rated as quacks, till the present time, we find it in continual use as a tooth-saver.

Its first advent was under very unfavorable circumstances. All that the leaders in the profession could say against it was said. The

---

[1] Read before the Harvard Odontological Society, April 28, 1892.

controversy was carried into the press, producing a public feeling in opposition to it. It was denounced by dental societies even to the expulsion of all members who would not sign a pledge not to use it.

In a few years this pledge was rescinded, but the society was unable to survive its misdeeds and disbanded.

In 1859 the editor of the *Dental Cosmos* said, "We did not suppose that any one would venture to publicly advocate it after so many able men have failed to sustain it."

*Preparations used.*—The early fillings were made from silver coin; they saved the teeth well, but turned them dark.

Cadmium was introduced by Dr. Evans, of Paris, and was first discarded by him; it caused the dentine to become soft.

It is not necessary to name the different metals or the proportion in which they have been used, only to remark that as a rule the best tooth-savers have been those alloys that turned dark.

During the past few years copper amalgam—not copper in combination with other metals (whose merit has been long recognized), but copper and mercury—has been used extensively. It has been discussed in dental societies, manufactured and sold with profit by the dental depots, and used without profit by the dental profession.

It may be called one of the black spots in our history. Let us now look at a few of the causes which aided in the introduction of amalgam.

1. A large class of teeth could not be saved by non-cohesive gold fillings. These teeth were extracted. It was found that amalgam did save these teeth, many of them that had been filled several times with gold.

2. The introduction of cohesive gold and its extensive use marked another dark spot in our history. Because one piece of gold would stick to another and contour could be restored, many monuments were built which brought ruin to the teeth, disappointment to the patient, and chagrin and loss of reputation to the operator. Aside from all this, the damage to the patient's health by the long operations, the dread and fear produced in the public mind concerning our calling, are results the magnitude of which we cannot estimate.

3. The introduction of cement and the failure of the same also prepared the way for amalgam. What wonder that some people should have adopted an idea that filling teeth did not save them, when we consider the various cements used, which were carefully prepared by our dental manufacturers, quickly inserted by the dentist, and as promptly dissolved by the fluids of the mouth.

All of these failures prepared the way for a more extended and proper use of amalgam.

The New Departure advocates, in 1879, began to emphasize the use of plastics, with good results.

*Uses.*—Amalgams have been found useful in the following cases:

1. In temporary teeth.
2. In setting crowns.
3. In large and inaccessible cavities.
4. In patching gold fillings.
5. In compound fillings especially. (Dr. Clapp's method.)
6. In compound fillings combined with oxyphosphate.

One of our ablest practitioners recently told me that without amalgam one-third of the teeth he now saved would be lost.

I saw yesterday a large filling, crown and distal, in a lower molar that had preserved the tooth perfectly for twenty-four years, the pulp was dead at the time of filling, and through a perforation in the gum at the point of bifurcation of the roots blood came very freely at the time of filling. I think no other material would have served as well.

To many the color is a serious objection; this is less so than formerly, and now, by the use of Dr. Clapp's combination fillings, may be avoided. The change of color which occurs in some cases does not give us an artistic filling, but does not apparently interfere with its preservative action. Change of shape does not always occur, and in many cases may be due to improper shaping of the cavity, in others to the amalgam being poorly mixed; the alloy should be thoroughly united with the mercury in a mortar, not mixed like water and sawdust.

Who is responsible for that abominable impression in the minds of most patients that gold is the best material to fill teeth with? Who has, at the expense of time, money, and nervous force inserted these large gold fillings which have too often proved failures? Do you call such things evidence of skill? We maintain that the length of an operation is not evidence of it. The insertion of a gutta-percha or amalgam filling in an inaccessible place often requires more skill than the filling of a large cavity with gold. "Man makes the material, not material the man." Is there anything worse for a man who wishes to be lazy and yield to the easy way of doing an operation regardless of what is best for the patient, than the unrestricted use of the oxyphosphate fillings, which stick in a cavity where an amalgam would fall out. Cavities must be shaped, at least, to hold an amalgam filling. One of the most brilliant operators our school has ever graduated confessed to me that the use of oxyphosphate and gutta-percha fillings unfitted him for extensive gold operations.

We have had our experience with copper amalgam. We have seen very excellent results, some passable and others that were bad. It does in some cases dissolve at the cervical margin, and any filling, whatever may be the preservative action of its several component parts, if, when in combination, they easily dissolve, we cannot consider it a safe material to use. One other objection arising from this same defect is that, although inserted when the condition of the mouth may be such that the fillings do excellent service, changes may occur in the health of the patient which make the filling an undesirable one. We have, therefore, discarded it.

The question of its effects on the system is a serious one. If it does poison our patients we must not use it, better that the teeth be lost than that the whole body should suffer harm. For sixty years the cry has been salivation. As Goliath of Gath brought terror to the hearts of the Israelites, so this monster mercury, which by injudicious use had wrought ravages in the ranks of medical patients, has brought alarm to this generation, because amalgam fillings contain mercury. Probably ninety per cent. of people having teeth filled have at some time had an amalgam. We have here to-night an accumulated practice of what would be to one man at least a century of experience. How many cases did you ever see that were salivated? How many that you could positively say were in any way injured by an amalgam filling? How many poisonous results did you ever observe from the mixing of amalgam fillings in the hand?

The early objections to amalgam was that it produced alveolar abscess, exostosis, necrosis, and might cause ptyalism. In 1872 we had a case reported in the *Dental Cosmos* of death from an amalgam filling, but the verdict could have been gutta-percha as far as symptoms were concerned, alveolar abscess being the probable cause.

No testimony of salivation from amalgam which occurred previous to 1870–75 should have any weight, as the results claimed to have been caused by the mercury may have come from pyorrhœa alveolaris, which disease was not generally recognized previous to that time. A case in point was given me a few days ago by a brother practitioner.[1]

A patient was being treated for syphilis by his physician; mer-

[1] Transactions of the American Dental Association, 1884, p. 68.

cury was used, causing salivation, which did not cease on the remedy being discontinued for three months. By the advice of his physician he consulted a dentist, who found the usual deposit of black tartar under the gum, which he removed. In two weeks the patient went back to his physician cured of salivation, and the mercury treatment being continued, salivation did not occur again. The experiment of Dr. Bogue, 1875, proves that no mercury is dissolved by the acids in the saliva.

A few weeks ago in discussing this subject in this Society, it was regretted that the dental and medical profession could not come to a better understanding in this matter. Such an understanding which would give us the truth would be one greatly to be desired, but we have different points of viewing this question. A physician's diagnosis on a dental case is, as a rule, unreliable. The profession have not been interested in the etiology and treatment of these cases. If we had a chair of dentistry in each medical school it would help somewhat, but even under these circumstances we cannot hope to harmonize dentistry, as now practised, with that branch of medical science that dispenses so-called high potencies.

Poisoning from corrosive sublimate is charged upon us by the medical profession. (Read, S. C., 1874–79, 213.)

Dr. Frances, of New York, reports a case where a patient of his was told by her physician that the poison in the fillings antagonized the remedies he was giving her. She believed him, went to her dentist, who discovered five gutta-percha and two tin fillings.

Such results follow where mercury is not present, but what disastrous effects may be realized from the vapor of mercury coming into the sleeping-apartments of people who have several large mirrors therein!

We cannot now say that harm does not occur, but can you assert that the quantities of zinc oxide from the plastic fillings taken into the system do not cause cancer? I had a friend who died of cancer some few months after having an oxyphosphate filling inserted. May not the free acid in an oxyphosphate filling cause disease? You may smile at the idea, but with the concurrence of a very few physicians a dentist could cause great fear to fall upon the people who have these fillings inserted in this city of ours.

I had a patient who was attacked with iritis; he had had several attacks before; was treated by a regular practitioner, but not cured. A friend mentioned his case to a Christian scientist, who treated him without seeing him, his eye was cured almost immediately, though in previous attacks he had been confined to a dark room for two months. Is regular medicine, therefore, a failure? A patient has two or three small amalgam fillings removed. Seen a year later, she says she has had better health the past winter than the previous year; amalgam fillings are, therefore, poisonous.

The profession has waited sixty years for the proof that an amalgam filling can produce disease. It still waits for it. Tons of this material are used annually in this country alone. Is there *one* cavity which can be better filled by its use than by the use of any other material? And we have tied our hands so that we cannot use it, we have failed where we might have succeeded, and the ideal practice is not ours.

